# GSH-Independent Induction of ER Stress during Hypoglycaemia in the Retinal Cells of Mice

**DOI:** 10.3390/jcm10112529

**Published:** 2021-06-07

**Authors:** Daria Fresia, Enrica Cannizzaro, Angelica Borgo, Raphaël Roduit

**Affiliations:** Department of Ophthalmology, University of Lausanne, Jules-Gonin Eye Hospital, 1002 Lausanne, Switzerland; daria.fresia@fa2.ch (D.F.); enrica.cannizzaro@fa2.ch (E.C.); angelica.borgo@fa2.ch (A.B.)

**Keywords:** hypoglycaemia, diabetic retinopathy, neurodegeneration, glutathione (GSH), endoplasmic reticulum (ER) stress, ERAI (ER stress-activated indicator) mice, buthionine sulfoximine (BSO), glutamate cysteine ligase (Gclm)-KO mice

## Abstract

Glucose is one of the most important metabolic substrates of the retina, and glycaemic imbalances can lead to serious side effects, including retinopathy. We previously showed that hypoglycaemia induces retinal cell death in mice, as well as the implication of glutathione (GSH) in this process. This study aimed to analyse the role of low glucose-induced decrease in GSH levels in endoplasmic reticulum (ER) stress. We cultured 661W photoreceptor-like cells under various glucose conditions and analysed ER stress markers at the mRNA and protein levels. We used the ERAI (“ER stress-activated indicator”) mouse model to test ER stress in both ex vivo, on retinal explants, or in vivo, in mice subjected to hypoglycaemia. Moreover, we used buthionine sulfoximine (BSO) and glutamate cysteine ligase (Gclm)-KO mice as models of low GSH to test its effects on ER stress. We show that the unfolded protein response (UPR) is triggered in 661W cells and in ERAI mice under hypoglycaemic conditions. Low GSH levels promote cell death, but have no impact on ER stress. We concluded that low glucose levels induce ER stress independently of GSH levels. Inhibition of ER stress could prevent neurodegeneration, which seems to be an early event in the pathogenesis of diabetic retinopathy.

## 1. Introduction

Diabetic retinopathy (DR) is the leading cause of vision impairment in working-age adults [1]. However, despite years of investigation, mechanisms underlying the development and progression of diabetic retinopathy remain elusive, and therapies developed to counteract vascular damage are still inefficient [2]. Since its discovery, DR has been identified as a vascular disease caused by the increased production of reactive oxygen species (ROS) in pericytes and capillary endothelial cells due to chronic hyperglycaemia [3,4]. However, an increasing body of evidence suggests that neurodegeneration is an early event in the pathogenesis of diabetic retinopathy, which could precede the development of microvascular abnormalities [5,6]. In fact, neural apoptosis and reactive gliosis, the hallmarks of retinal degeneration, have already been reported in diabetic patients without microcirculatory abnormalities [7,8]. In addition, ROS production and oxidative stress have been shown to be central events in the development of DR [9,10].

The role of endoplasmic reticulum (ER) stress in neurodegeneration in DR has been clearly established, and impairment of ER homeostasis has been found in photoreceptors [11], retinal pigment epithelial cells [12], and Müller glial cells [13] using in vitro and in vivo models of diabetes. ER is the primary organelle responsible for protein folding, maturation and trafficking [14] and ER homeostasis is central to any cell. In addition, redox impairment has been observed under several stimuli, including glucose deprivation [15]. Under conditions of ER stress, the unfolded protein response (UPR) is activated. UPR is controlled by three ER-resident transmembrane proteins that are attached to the Hsp70-type chaperone binding immunoglobulin protein (BiP): inositol-requiring enzyme (IRE1), protein kinase R (PKR)-like endoplasmic reticulum kinase (PERK), and activating transcription factor 6 (ATF6). After being detached from BiP, they activate their downstream effectors: ATF6 is cleaved at the Golgi, PERK phosphorylates eukaryotic translation initiation factor (eIF2α) to slow down normal translation, while X-box binding protein 1 (*Xbp-1*) is unconventionally spliced by IRE1. Their function is to increase the levels of ER protein folding enzymes and chaperones, along with the degradation of accumulated proteins. If the protein folding and degradation capacity of ER is overwhelmed by a sustained stress, UPR promotes cell death by increasing the translation of CCAAT-enhancer-binding protein homologous protein (CHOP) accompanied with the activation of pro-apoptotic components of the B-cell lymphoma 2 (BCL-2) family (for review, see [16]). It has been recently proposed that modulating UPR could have a protective role in retinal cells, and BiP gene delivery has been shown to ameliorate ER stress in the retina of mice [17,18]. Recently, Ikesugi et al. demonstrated that ER stress is involved in the cellular mechanism of DR by showing that glucose fluctuation, in particular hypoglycaemia, activated UPR in pericytes [19].

Glutathione (GSH) is the most abundant hydrophilic antioxidant that protects cells against exogenous and endogenous toxins [20]. GSH scavenges free radicals, ROS, and reactive nitrogen species (RNS) directly and indirectly through various enzymatic reactions. Synthesis of GSH from its constituent amino acids (glutamate, cysteine, and glycine) is a two-step reaction that requires ATP. The γ-glutamylcysteine synthetase (GCS) catalyses the first and rate-limiting reaction, while the second is catalysed by glutathione synthetase (GS). A decrease in GSH biosynthesis, as well as its depletion upon being used up for detoxification, an increased breakdown, or a failure in its regeneration from its oxidised form GSSG, lead to a decrease in cellular GSH levels and contribute to oxidative stress [21]. ER, similar to other organelles, has its own pool of GSH; however, despite its central role in ER homeostasis, very few studies exist on GSH quantification and the relationship between ER and cytosolic GSH [22,23].

In the diabetic retina, photoreceptors are the major source of oxidative stress owing to their high energy demand and light exposure [24,25]. As a consequence of increased oxidative stress, antioxidant defenses are decreased in the diabetic retina [26]. Photoreceptors are the most abundant cells of the retina and have a unique function in the body: absorbing light and converting it into electrical energy that results in sight [27]. As photoreceptors have no glycogen reserves [28] and do not require insulin for glucose uptake, they are directly damaged in case of glucose depletion [29].

The effects of acute hypoglycaemia on the retina of subjects with or without diabetes were investigated for the first time in 2011 by Khan et al. They found decreased central retinal function in all subjects with diabetes after acute hypoglycaemia, hypothesising that during this period cells are unable to meet their metabolic demands due to an insufficient glucose supply [30]. Hypoglycaemia is not to be ignored for diabetic patients, with the average type 1 diabetic patient estimated to suffer two episodes of symptomatic hypoglycaemia per week, leading to thousands of such episodes over a lifetime, and at least one episode of severe hypoglycaemia per year [31]. Hypoglycaemia is less frequent in the first few years of treatment for type 2 diabetes; however, the risk increases substantially with the progression of the disease [32].

The harmful effect of hypoglycaemia was studied in mice, and in 2011, we observed an increase in oxidative stress in retinas collected from mice that underwent a 5 h hypoglycaemic clamp and in 661W photoreceptor-like cells that were cultured at low glucose levels. As a consequence of increased oxidative stress, GSH levels were found to decrease [33]. Moreover, microarray analysis of retinas collected 4 h and 48 h after the clamp showed broad changes in the expression of genes involved in lysosomal function, GSH metabolism, and apoptotic pathways [34].

Therefore, in this study we aimed to investigate the effect of ER stress in vitro using 661W photoreceptor cells cultured under low glucose conditions and in vivo using ER-stress associated indicator (ERAI) mice subjected to a 5 h hypoglycaemic state. Additionally, we investigated the correlation of a decrease in GSH levels with ER-stress related effects by treating 661W cells with buthionine sulfoximine (BSO), an inhibitor of GSH synthesis and using the glutamate cysteine ligase (Gclm) knockout mice as a model of low GSH content.

## 2. Materials and Methods

### 2.1. Mouse Lines

This study was performed as per the guidelines of the Association for Research in Vision and Ophthalmology (ARVO) for the use of animals in ophthalmic and vision research and was approved (permit number VD3155 and VD3531) by the veterinary service of the State of Vaud (Switzerland). The ER-stress associated indicator (ERAI) mouse model was described by Iwawaki et al. and allows to detect a GFP signal when UPR is activated in cells. In this transgenic mouse, GFP is a marker for *Xbp-1* splicing in vivo during ER stress [35]. Glutamate-cysteine ligase modifier subunit (Gclm) knockout mouse model has been characterised elsewhere [36] and is used as a model with decreased GSH content. Wild-type C57BL/6 mice (WT) were purchased from Charles River Laboratories (Les Oncins, France) and bred for maintenance in our animal facility. Animals were kept in a 12 h light/12 h dark cycle with unlimited access to food and water.

### 2.2. Cell Culture Conditions

Retinal explants were isolated from 15-day-old mice and cultured for 24 h on a Costar Transwell Permeable Support (Corning, #3412) in R-16 complete medium (#074-90743A Life Technologies Inc, Carlsbad, CA, USA) supplemented with NaHCO_3_ at a final concentration of 32.5 mM (#71628 Fluka Thermofisher Scientific, Waltham, MA, USA) and a mixture of several growth factors (Sigma Aldrich, St. Louis, MO, USA) as described previously [37]. Subsequently, the explants were cultured for 48 h under different glucose conditions as described previously by Emery et al. [33]. Retinal explants were then collected, fixed in 4% paraformaldehyde for 20 min, and then included in Yazzulla (30% egg albumin and 3% gelatine in PBS) after a sucrose (#1.07687.1000 Merck Millipore, Darmstadt, Germany) gradient (2 h sucrose 10%, then 2 h sucrose 20% and 2 h sucrose 30%). The explants were subsequently cut and stained for GFP (1:1000, Abcam #290, Cambridge, UK) and GFAP (1:500, #Z0334, Dako, Glostrup, Denmark). The 661W photoreceptor cell line was maintained in routine culture in Dulbecco’s modified Eagle’s medium (DMEM, Thermo Fisher Scientific, Waltham, MA, USA, #11330-032) as described previously [38]. To evaluate low glucose conditions, cells were first synchronised for 24 h in DMEM supplemented with 1% foetal bovine serum (FBS, Pan-Biotech GmbH, Aidenbach, Germany, #P30-3306), and then switched to glucose-free DMEM (Pan-Biotech GmbH, Aidenbach, Germany, #P04-01548s1) supplemented with 1 mM or 25 mM glucose (#G8644, Sigma Aldrich, St. Louis, MO, USA) for 24 h. Thapsigargin 2.4 M (#T9033, Sigma Aldrich, St. Louis, MO, USA) was used as a positive control to induce ER stress. To modulate GSH levels, cells were firstly synchronised for 24 h in DMEM #11330-032 with 1% FBS, then treated with glucose-free DMEM supplemented with 25 mM glucose, along with 2 μM, 5 μM, or 10 μM of L-buthionine sulfoximine (BSO) (#B2515, Sigma Aldrich, St. Louis, MO, USA). Human embryonic kidney 293T (HEK293T) cells were maintained in DMEM supplemented with 10% FBS and 1% penicillin/streptomycin (#P4485 Sigma Aldrich, St. Louis, MO, USA).

### 2.3. RT-PCR and qPCR

RNA was isolated using TRI reagent (#T9424, Sigma Aldrich, St. Louis, MO, USA) and chloroform (#1024451000, Merck Millipore, Darmstadt, Germany). Briefly, 500 µL of TRI Reagent was added directly to 661W cells that were pre-washed with Hank’s balanced salt solution (HBSS) (#H6648, Sigma Aldrich, St. Louis, MO, USA). Cells were then scraped and collected into a tube and left at 25 °C for 3 min. Then, 200 µL Chloroform was added to the cells, mixed by inverting the tube briefly and incubated for 3 min at 25 °C. The cells were then centrifuged at 15,700× *g* at 4 °C for 15 min. An amount of 700 µL of ice-cold isopropanol (#1096341000, Merck Millipore, Darmstadt, Germany) was added drop by drop and mixed by inverting the tubes. The pellet, obtained by centrifugation for 10 min at 15,700× *g* at 4 °C, was then washed with 500 µL ethanol 75% in distilled water (#1023711000 Merck Millipore, Darmstadt, Germany), centrifuged for 5 min at 7600× *g* at 4 °C, and the RNA thus obtained was resuspended in ultrapure H_2_O and incubated at 60 °C for 10 min. Then, 1 µg of total RNA was used for cDNA synthesis according to the manufacturer’s instructions (High-Capacity cDNA Reverse Transcription Kit #4368813, Applied Biosystems, Foster City, CA, USA) in a Biometra Tadvanced thermocycler from Analytik Jena AG. One microgram of cDNA was used to detect various spliced forms of *Xbp-1* using GoTaq DNA polymerase (#M3001, Promega, Madison, WI, USA). The PCR amplification protocol was set to 10 min at 95 °C, followed by 1 min each at 95 °C, 59 °C, and 72 °C for 50 cycles, and final elongation at 72 °C for 10 min. cDNA (5 ng) was used for quantitative PCR amplification using the FastStart Essential DNA Green Master (#06924204001 Roche, Basel, Switzerland). Primers (Sigma-Aldrich) were used as described in Supplemental Table S1. The qPCR analysis for multiple replicates was performed using LightCycler 96 (Roche Diagnostics, AG), with the following program: a pre-incubation of 5 min at 95 °C, followed by a 3-step amplification (95 °C, 60 °C, and 72 °C for 10 s each; 45 cycles) and a 3-step melting (95 °C for 10 s, then 65 °C for 1 min, and 97 °C for 1 s). The cooling step was performed for 30 s at 37 °C.

### 2.4. Western Blotting Analysis

Cells were collected in RIPA buffer (50 mM Tris (pH 8.0), 150 mM NaCl, 1% NP-40, 0.5% sodium deoxycholate, 0.1% SDS) supplemented with 1× phosphatase inhibitors cocktails (#P0044 and #P5720, Sigma Aldrich, St. Louis, MO, USA) and 1× protease inhibitors (#P8340, Sigma Aldrich, St. Louis, MO, USA), and cell lysates were obtained with three freeze–thaw cycles of 20 min each and centrifugation at 4 °C at 15700× *g* for 20 min. Total protein quantification was performed on cell lysates with Pierce BCA protein assay kit (#23235, Thermo Fisher Scientific, Whaltam, MA, USA). Fifty micrograms of total proteins were separated by SDS-PAGE, then electrically transferred to PVDF membranes (#IPFL00010, Merck Millipore, Darmstadt, Germany) and incubated with anti-GRP78/BiP (1:1000, Cell Signalling Technology, Danvers, MA, USA, #3183), anti-GADD153/CHOP (1:500, Novus Biologicals, Littleton, CO, USA #NB600-1335), anti-ATF4 (1:1000, BioLegend, San Diego, CA, USA, #693901), and anti-ATF6 (1:200 Boster Biological Technology #BOS- PA1011-1) primary antibodies overnight at 4 °C with mild shaking. β-Actin (anti-β-actin, 1:500, LI-COR Biosciences, Lincoln, NE, USA, #926-42210) or α-tubulin (anti-α-tubulin, 1:2500, Sigma Aldrich, St. Louis, MO, USA #T6074) were used for normalisation. The secondary antibodies IrDye 800CW goat-anti mouse (#926-32210), IrDye 800CW goat-anti rabbit, (#926-32211), IrDye 680RD goat anti-mouse (#926-68070), and IrDye 680 CW goat anti-rabbit (#926-68071) (1: 15,000, LI-COR Biosciences, Lincoln, NE, USA) were used as appropriate to detect protein expression with the Azure Biosystems 600 machine (Axon Lab).

### 2.5. Endoplasmic Reticulum (ER) Isolation

Three T-150 flasks of confluent 661W cells were used for ER isolation. Briefly, 2.5 × 10^6^ cells were seeded per T-150 flask and incubated in DMEM with 1% FBS for 24 h. Then, cells were treated for another 24 h with DMEM without glucose, supplemented with 25 mM or 1 mM glucose, with or without 25 mM BSO (10 µM). At the end of the treatment, the cells were detached using trypsin and centrifuged at 1480× *g* for 5 min. Cells were then resuspended in 1 mL of ice-cold 250-STMDPS buffer (250 mM sucrose, 4 mM MgCl_2_, 50 mM Tris-HCl, pH 7.4) supplemented with 1x protease inhibitors (#P8340, Sigma Aldrich, St. Louis, MO, USA), and each sample was homogenised in a 2-mL Kimble Dounce homogeniser (#D8938 Sigma Aldrich, St. Louis, MO, USA) placed on ice for a minimum of 20 min. The opaque solution thus obtained was transferred into clean tubes and centrifuged for 15 min at 800× *g* at 4 °C. After centrifugation, the supernatant was collected and the pellet was re-processed with 500 µL of 250-STMDPS buffer with protease inhibitors as mentioned above. After homogenisation for 15 min and centrifugation at 800× *g* for 15 min at 4 °C, the supernatant obtained, along with the supernatant previously collected, was transferred to a clean tube and centrifuged at 6000× *g* for 15 min at 4 °C. The mitochondria- containing pellets were discarded and the supernatant was collected and ultracentrifuged for 1 h at 100,000× *g* in a swing-bucket ultracentrifuge, to let the ER membranes precipitate. At the end of the ultracentrifuge, the supernatant was aspirated and discarded, and the pellet containing the ER protein fraction was resuspended in 20 µL of RIPA buffer for protein analysis and GSH measurement.

### 2.6. GSH Analysis

The 661W photoreceptor cells were cultured as described above, and 20 μg of the total protein lysate was used to measure GSH by colorimetric assay according to the manufacturer’s protocol (glutathione detection kit #ADI-900-160, Enzo Life Sciences, Farmingdale, NY, USA). Briefly, 20 μg of total protein or 5 μg of ER-protein was precipitated with ice-cold metaphosphoric acid (5% final concentration; #239275 Sigma Aldrich, St. Louis, MO, USA) in a total volume of 20 µL. After precipitation and centrifugation for 15-min at 4 °C at 15,700× *g*, the supernatant was collected and diluted 100-fold with RIPA buffer. After 15 min of centrifugation at 4 °C at 15,700× *g*, 10 µL of supernatant was added to a clear 96-well half-area plate (#675101 Greiner Bio-One, Kremsmünster, Austria) with 15 µL of assay buffer. A four-point standard curve was generated by diluting GSSG in the assay buffer to obtain a final concentration of 100, 50, 25, and 12.5 pM/well. Then, 75 µL of reaction buffer, with reductase enzyme at a concentration of 1.25 μL/mL, was added to each well. After the addition of the reductase enzyme, the kinetics of the reaction were evaluated every 1 min for a duration of 10 min using a microplate reader (EnVision 2105, Perkin Elmer, Waltham, MA, USA).

### 2.7. Tunicamycin Injections

Two-month-old ERAI mice were injected with tunicamycin (TU) (#SML1287, Sigma Aldrich, St. Louis, MO, USA) intraperitoneally (50 ng/kg) or intravitreally (10 ng/kg), respectively, after being anaesthetised with a ketamine/xylazine mixture. DMSO was used as a negative control and was injected in the control animals intraperitoneally and in the contralateral eye intravitreally. Organs were collected 2 days after intraperitoneal injections, fixed with 4% paraformaldehyde (#P5148, Sigma Aldrich, St. Louis, MO, USA), embedded in paraffin, and then sectioned and stained for GFP (1:1000, Abcam #290, Cambridge, UK). Isolated retina from injected intravitreally eyes were collected after 4 h and analysed by Western blot analysis after protein extraction.

### 2.8. Terminal dUTP Nick End-Labelling (TUNEL) of Fragmented DNA

In situ detection of cell death in 661W cells, cultured for 24 h under different glucose conditions, was performed by the TUNEL assay as per the manufacturer’s instructions (In Situ Cell Death Detection Kit–TMR Red #12156792910 Roche Applied Science, Rotkreus, Switzerland). For each condition, apoptotic cells were visualised under a Leica DM6B microscope equipped with a DFC9000GT camera. (Leica Microsystems, Wetzlar, Germany) using appropriate filters. TUNEL-positive cells from six different fields per condition (*n* = 3) were counted using ImageJ software, version 1.53j (Rasband, W.S., ImageJ, U.S. National Institutes of Health, Bethesda, MD, USA, https://imagej.nih.gov/ij/, accessed on 7 June 2021).

### 2.9. Immunostaining

Slides of retinal explants as well as tissues injected with tunicamycin were blocked for 45 min with 10% normal goat serum (NGS #Y0907, Dako, Glostrup, Denmark) and 0.1% TritonX-100 (#1.08603.1000, Merck Millipore, Darmstadt, Germany) in PBS, and incubated with primary anti-GFP antibody (1:1000, Abcam #290, Cambridge, UK) for 1.5 h at 4 °C, then washed and incubated with Alexa Fluor antibody (#GAR488 1:2000, ThermoFisher Scientific) for 1 h. After washing, the slides were counterstained with DAPI (#D9542 Sigma Aldrich, St. Louis, MO, USA) for 10 min and covered with citifluor AF1 (Glycerol/ PBS solution #E17970 Science Services) and coverslips (Menzel-Glaser, 24 × 60 mm BB02400600A113MNZ0, Thermofisher Scientific). Images were acquired using a fluorescence microscope (Leica DM6B microscope equipped with a DFC9000GT camera. (Leica Microsystems, Wetzlar, Germany).

ERAI Retinal explants slide were pre-treated for 10 min with 0.5% Triton in PBS à RT, then washed 3× with PBS and blocked for 1 h with 5% NGS, 1% BSA (Albumin Fraction V, #A1391,0100 Applichem) and 0.1% Triton in PBS at room temperature. The first antibodies against GFAP (#Z0334, 1:500, Dako, Glostrup, Denmark) or against cleaved-caspase3 (#9661, 1:200, Cell Signaling Technology, Danvers, MA, USA) were then added in blocking buffer, then left overnight at 4 °C. After 3× washing in PBS, the secondary antibodies Alexa Fluor (#GAR488 or #GAR633, 1:2000, ThermoFisher Scientific) for 1 h 30 min at 22 °C. After 3× washing with PBS, nuclei were stained with DAPI, then washed again 3× with PBS and mounted with Citifluor and coverslips.

### 2.10. Hyperinsulinemic Clamp

An indwelling catheter (Becton Dickinson AG, Basel, Switzerland) was inserted into the femoral vein of 10 isoflurane-anesthetised mice (4 mice for the euglycaemic group and 4 mice for the hypoglycaemic group and 2 sham operated). The animals were allowed to recover for 14 days. After a 5 h fasting period, awake and freely moving mice were subjected to 5 h of either a hyperinsulinaemic/hypoglycaemic clamp or a hyperinsulinaemic/euglycaemic clamp, as described previously [39]. Mice were sacrificed 4 h post-clamp, and eyes were either collected to be embedded in paraffin, sectioned, and stained for GFP (1:1000, Abcam #290, Cambridge, UK).

### 2.11. Statistical Analysis

All results are expressed as mean ± SEM of the indicated number of experiments. Data were statistically analysed using Prism 6.0. Each group of data was tested for normality of distribution using the Shapiro–Wilk test. In the case of normal distribution, we used Welch’s ANOVA test (one-way ANOVA with unequal variances) followed by a post hoc Tukey–Kramer test to compare the differences. When the distribution was not normal, we used a Kruskal–Wallis test (non-parametric analogue of one-way ANOVA) to compare the different treatments. The results were considered statistically significant at a *p*-value of *p* < 0.05.

## 3. Results

### 3.1. Expression of UPR-Related Genes Is Increased in 661W Cells under Hypoglycaemic Stress

**Figure 1:**
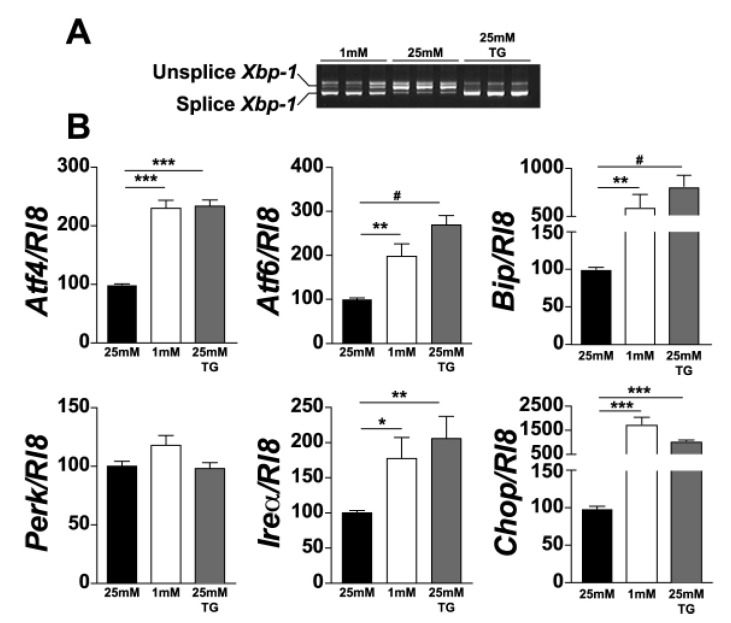
Low glucose condition increases *Xbp-1* mRNA splicing and expression of ER stress genes in 661W cells. After treatment of 661W photoreceptor cells with ER-stress activators, *Xbp-1* mRNA splicing was evaluated by PCR (**A**). UPR-associated gene expression was evaluated by qPCR, normalised with *Rl8* expression (**B**). Results are expressed as mean ± SEM of three experiments (*n* = 7; * *p* < 0.05, ** *p* < 0.005, *** *p* < 0.0001, # *p* < 0.00001).

**Figure 2:**
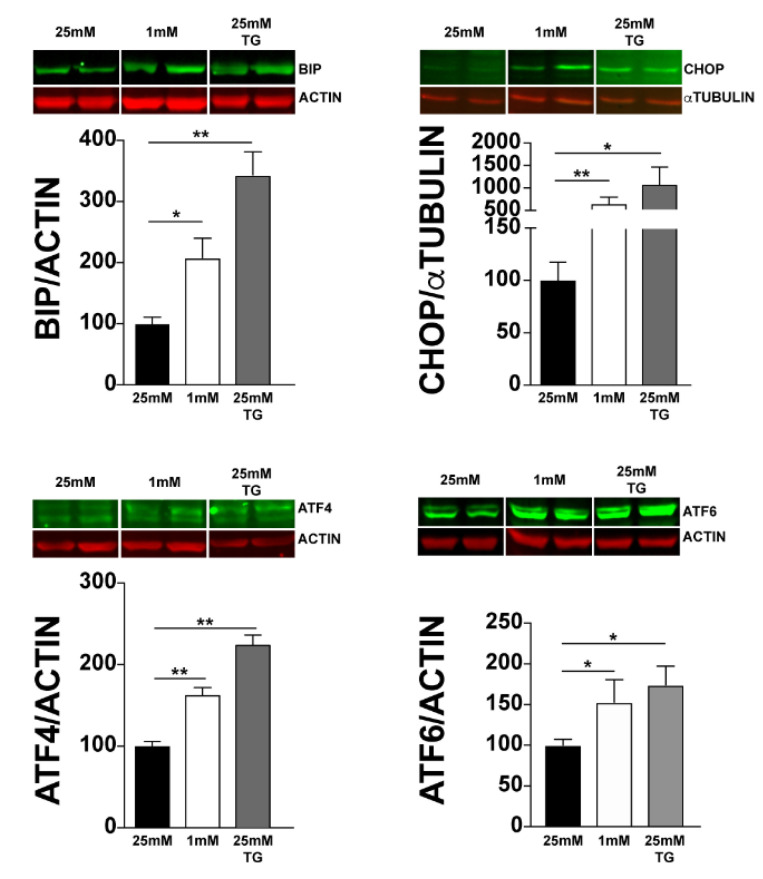
Effect of low glucose conditions on the expression of ER stress proteins. Protein levels from 661W cell lysates were evaluated using Western blotting analysis, with β-actin and α-tubulin for normalisation. Results are expressed as mean ± SEM of three experiments (*n* = 6; * *p* < 0.05, ** *p* < 0.009).

Since UPR is triggered by glucose deprivation and photoreceptors have the highest demand for energy among retinal cells, we investigated whether UPR is triggered in 661W photoreceptor cells under low-glucose conditions. We analysed the expression of the genes involved in UPR in 661W cells cultured at 25 mM or 1 mM glucose for 24 h. Thapsigargin (TG), a compound that activates UPR, was added to cells cultured at 25 mM, as a positive control. We observed the induction of *Xbp-1* splicing under low-glucose conditions (Figure 1A). In addition, after normalisation by *R*l8, we also observed an increase in the expression of genes involved in all the three arms of UPR. Indeed, we observed a significant increase in the expression of *Bip* (5-fold) and *Chop* (15-fold). This increase was smaller, but significant, for *Atf*4, *Atf*6, and *Ire*1*α* (Figure 1B). We did not detect any significant increase in *Perk* expression either under low-glucose conditions or after TG treatment. Moreover, we confirmed UPR activation at the protein level using Western blot analysis with either actin or α-tubulin for normalisation. We observed a significant increase in *BIP*, *CHOP*, *ATF4*, and *ATF6* proteins (Figure 2). These results clearly show the activation of UPR in 661W photoreceptor cells under conditions of low glucose concentration.

### 3.2. UPR Is Also Triggered Ex Vivo and In Vivo under Low Glucose Conditions

**Figure 3:**
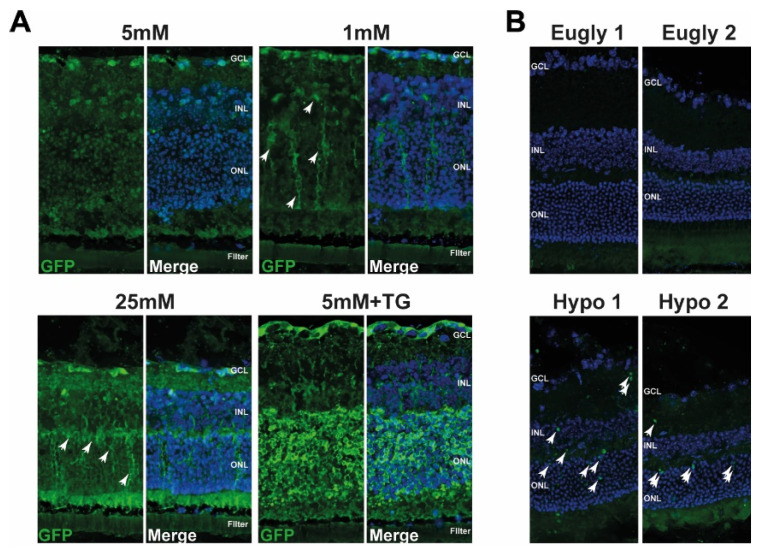
*Xbp-1* splicing is activated in ERAI^tg/+^ retinal explants after 48 h and after a 5 h hypoglycaemic clamp. (**A**) Confocal images showing retinal explants from ERAI^tg/+^ mice cultured at different glucose conditions (1 mM, 25 mM or 5 mM with or without TG). White arrows indicate GFP-positive cells and nuclei are counterstained with DAPI. Results are representative of 2 retinal explants per condition. (**B**) Endogenous GFP detection (white arrows) in the retina of mice subjected to a hypoglycaemic (Hypo1 and Hypo2) or euglycaemic (Eugly1 and Eugly2) clamp; nuclei are counterstained with DAPI. Eyes were isolated 4 h after the end of the clamp. Results are representative of 4 retinas for each group (Hypo1 and Hypo2 are two different mice, Eugly1 and Eugly2 are two different mice). GCL: ganglion cell layer, INL: inner nuclear layer, ONL: outer nuclear layer.

To further characterise UPR induction, we used the ER-stress associated indicator (ERAI) mouse model [35] to examine UPR ex vivo in isolated retinas cultured at low glucose conditions and in vivo during hypoglycaemia. To verify the validity of this model, we injected ERAI mice intraperitoneally with tunicamycin (TU) or PBS as a control. We observed an increase in GFP-positive cells in the liver and retina when the animals were treated with TU, an ER stress activator (Supplemental Figure S1A). Moreover, intravitreal injection of TU, with PBS injection in the contralateral eye as a control, showed an increase in GFP signal in all treated eyes (Supplemental Figure S1B). These results suggest that ERAI mice are suitable for studying UPR in the retina. We then isolated retinas from ERAI mice and cultured them at physiological (5 mM), high (25 mM), or low (1 mM) glucose concentrations. We observed a fairly strong GFP signal when the retina was cultured at high glucose with a similar, but less pronounced, pattern at low glucose (Figure 3A, white arrows); meanwhile, very few GFP-positive cells were detected at 5 mM glucose. The positive control of isolated retina cultured with 5 mM glucose in the presence of TG showed a very strong GFP signal (Figure 3A). Interestingly, most of the GFP-positive cells, but not all, were also positive for glial fibrillary acidic protein (GFAP), suggesting that these cells, in which ER stress was activated by low glucose culture conditions, were Müller cells (Supplemental Figure S2). We then investigated whether UPR was triggered by hypoglycaemia in vivo. ERAI mice were subjected to a 5 h hypoglycaemic clamp (Hypo), while the control euglycemic (Eugly) group received insulin and glucose infusions at the same time, as described previously [33]. Glycaemia was monitored during the entire clamp (data not shown) and retinas were collected 4 h after the end of the clamp. Figure 3B shows an increase in GFP-positive cells only in the retina of mice with hypoglycaemia, and not in the mice present in the euglycaemic group (Figure 3B); the two different retina isolated from hypoglycaemic (Hypo1 and Hypo2) and euglycaemic mice (Eugly1 and Eugly2) are representative of the four retina from each group. Both of these results showed that low glucose, either in cultured isolated retina or during a hypoglycaemic clamp, induces the activation of UPR in the retina.

### 3.3. GSH Decrease Is Not Involved in UPR Activation

**Figure 4:**
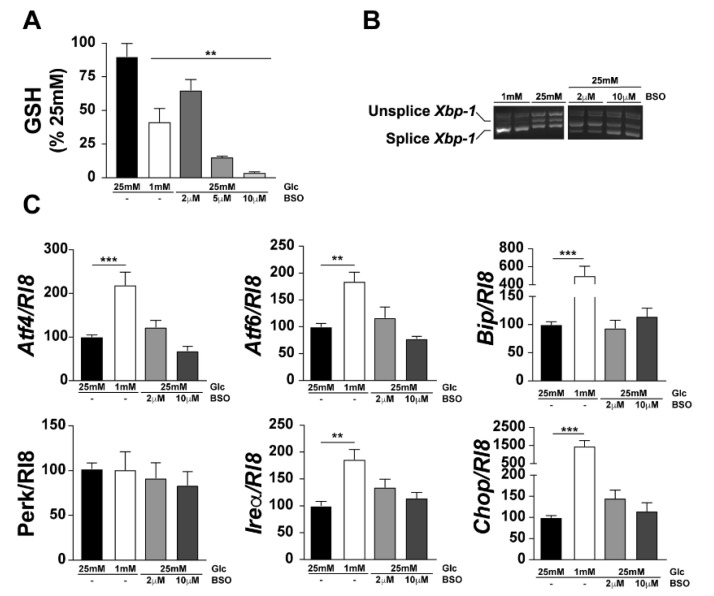
L-Buthionine-sulfoximine (BSO) administration decreases GSH in 661W cells but did not affect ER stress. The 661W cells were cultured with or without BSO (2, 5, or 10 μM). After treatment, proteins were extracted and quantified, and the GSH concentration was evaluated (**A**). *Xbp-1* splicing was evaluated by RT-PCR (**B**), and expression of other ER stress genes were evaluated by qPCR (**C**). Results are expressed as mean ± SEM of 2–3 experiments (*n* = 6–9; ** *p* < 0.005, *** *p* < 0.0001).

**Figure 5:**
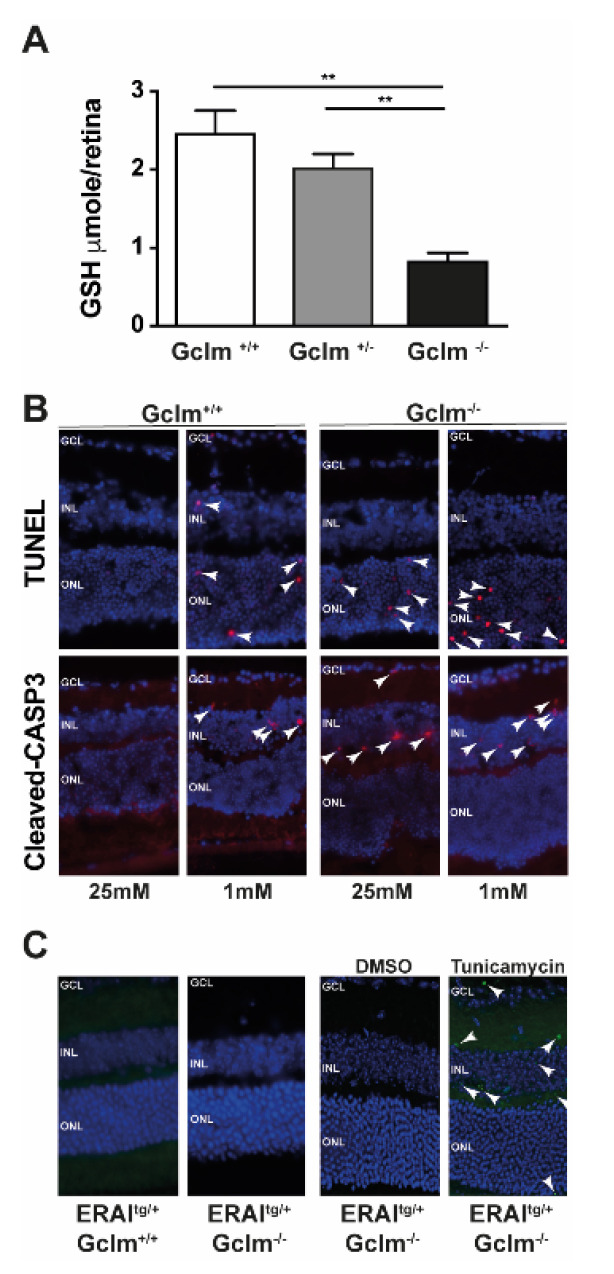
Cell death is activated in *Gclm* KO mice, but it is not related to UPR in ERAI^tg/+^/Gclm KO mice. (**A**) GSH quantification in retinas of adult *Gclm*^+/+^ (WT), *Gclm*^+/−^ and *Gclm*^−/−^ mice. Results are expressed as mean ± SEM of 4 retinas for *Gclm*^+/+^ (WT) and 3 retina for *Gclm*^+/−^ and *Gclm*^−/−^. ** *p* < 0.005. (**B**) TUNEL and activated-caspase3 staining of *Gclm^+/+^* and *Gclm^−/−^* mice retinas after 24 h of culture under conditions of high (25 mM) and low (1 mM) glucose concentration. White arrows show TUNEL-positive cells. Results are representative of 2 retina per condition. (**C**) GFP staining of adult ERAI^tg/+^/Gclm^+/+^ and ERAI^tg/+^/Gclm^−/−^ mice retinas. Intravitreal injection of Tunicamycin 10 ng/kg was used as a positive control and DMSO 0.2% in the contralateral eye was used as a negative control. White arrows show GFP-positive cells. Results are representative of 2 retina per condition. GCL: ganglion cell layer, INL: inner nuclear layer, ONL: outer nuclear layer.

We previously reported that modification of the GSH levels can modulate cell death [33]. In order to investigate the role of ER stress in the modulation of cell death, we hypothesised that a reduction in the GSH content in 661W cells might deregulate the ER redox state and trigger UPR. To test this hypothesis, we inhibited de novo GSH production in 661W cells to mimic the hypoglycaemia-induced decrease in GSH levels. The 661W cells cultured at low glucose (1 mM) concentration showed a decrease in the level of total GSH by 50%, as previously reported [33], while treatment with BSO of the cells cultured at 25 mM glucose showed a dose-dependent decrease in GSH levels with approximately 30% decrease at 2 µM BSO and a total disappearance of GSH at 10 µM BSO (Figure 4A). We then quantified the expression of UPR-related genes under low glucose conditions, and at the two different concentrations of BSO, and found no significant increase in *Xbp-1* splicing (Figure 4B) or in the expression of other UPR-related genes (Figure 4C). These results suggest that a decrease in GSH is not sufficient to induce ER stress in 661W cells. In addition, we measured GSH content specifically in the ER (GSH_ER_) at 1 mM and 25 mM glucose, in the presence or absence of BSO. Surprisingly, we observed no decrease in GSH_ER_ levels at 1 mM compared to 25 mM (data not shown), but observed a significant reduction in GSH_ER_ content after BSO treatment at 25 mM (89.7% ± 6.2%, *p* < 0.034, *n* = 3). Our next step was to confirm the results obtained in vitro using the *Gclm*^−/−^ mouse model. This knockout mouse model lacks the modulatory subunit of the GCL enzyme and shows 80% less GSH in the kidneys, liver, and plasma [36]. We characterised the GSH content in the retina of these mice and found significant decreases in GSH content in the *Gclm*^+/−^ mice (20%) and in *Gclm*^−/−^ (70%) mice compared with their wild-type littermates (Figure 5A). We found, as expected and previously shown in C57BL6/J mice, an increase in TUNEL-positive cells and in cleaved-Caspase 3 (Figure 5B, white arrows), when isolated retinas from *Gclm*^+/+^ mice were cultured under low glucose conditions, compared with the *Gclm*^+/+^ retinal cells cultured in the high (25 mM) glucose condition (Figure 5B). The decrease in GSH content (in *Gclm*^−/−^) was sufficient to induce cell death in cultured retinal explants at 25 mM, with a more pronounced effect at 1 mM glucose concentration (Figure 5B). To assess whether the GSH decrease observed in *Gclm*^−/−^ mice, could inherently trigger UPR activation in vivo, we crossbred ERAI mice with *Gclm*^−/−^ mice to generate a mouse line of ERAI^tg/+^/*Gclm*^−/−^ mice that displayed positive GFP staining upon the induction of ER stress. No GFP-positive cells were observed in ERAI^tg/+^/*Gclm*^−/−^ mice inherently, while injection of these mice with TU clearly showed an induction of ER stress compared to the injection of DMSO as a negative control in the contralateral eye (Figure 5C). These results confirmed that the decrease in GSH alone is a determinant of low-glucose-induced cell death but cannot induce UPR in vivo (Figure 5).

## 4. Discussion

Glucose is one of the most important source of energy for photoreceptors and is essential for photoreceptor survival [29]. Iatrogenic hypoglycaemia is a limiting factor in the glycaemic management of diabetes [32].

The effect of glucose deprivation was demonstrated in 2011 by Emery et al. in mice. Hypoglycaemia for five hours is harmful to mouse retinas as it causes an increase in ROS production, caspase-3 activation, and ultimately leads to apoptosis. GSH, the most important antioxidant defence against ROS in the cell, is also decreased after acute hypoglycaemia [33], and microarray analysis has shown a deregulation in the expression of some of the enzymes involved in GSH metabolism after acute hypoglycaemia in mice [34]. GSH has a central protective role against oxidative stress in the retina; when GSH is decreased by BSO injections, apoptosis increases in several cell types in mouse retinas [40] and in 661W photoreceptors [33].

During hypoglycaemia, photoreceptors are capable of promoting autophagy, as reported by Balmer et al. in 2013. In this study, they showed activation of autophagy in 661W cells and retinal explants after 24 h of hypoglycaemia through the activation of the AMPK/RAPTOR/mTOR pathway, as well as increased expression of LC3-II after 48 h in 661W cells. Interestingly, chemical inhibition of autophagy decreased LC3-II expression in parallel, with a decrease in the expression of the anti-apoptotic protein BCL-2, and consequent activation of caspase-3 leading to apoptosis [41].

Protein folding, maturation, and trafficking are the most important tasks of ER [42]. Although an oxidative environment is crucial to ensure proper protein folding, excessive accumulation of ROS can disrupt redox homeostasis in the ER, leading to the accumulation of misfolded proteins [43]. GSH contributes to removal of ROS from the ER of several cell types [44,45]. When ER stress is induced, the GRP78/BiP chaperone is detached from the three arms of UPR, promoting its activation. In the event of sustained UPR activation, cell death is initiated through the transcription of CHOP, which initiates caspase activation and apoptosis by deregulating the expression of Bcl-2. Interestingly, in cells where CHOP is constitutively expressed, there is a severe depletion in cellular GSH content and Bcl-2 transcription is dramatically decreased [46], and we previously observed a similar effect when 661W cells were exposed to low glucose conditions, including Bcl-2 and GSH decrease [41] and CHOP increase (this study).

Despite the presence of a majority of studies on the interplay between UPR and retinal diseases, the question of whether UPR is an orchestrated response that leads to cell death or it could be a part of retinal cell defence, remains poorly understood.

In this study, we focused on UPR in photoreceptors under hypoglycaemic conditions. Our results showed an increase in the expression of all the genes involved in the earliest phase of UPR, except for *PERK,* in 661W cells after 24 h of treatment with 1 mM glucose. PERK is an ER transmembrane protein which senses the accumulation of misfolded proteins and consequently promotes the deceleration of protein translation. Under ER stress, its activation is initiated by oligomerisation and autophosphorylation [47]. One possible explanation for this result could be that, following ER stress, the phosphorylation of PERK might be augmented rather than its expression. Consistent with this hypothesis, we found an increase in ATF4 (a downstream effector of PERK and eiF2α), both at the mRNA and protein levels (Figure 1 and Figure 2). Our results are in line with those of other studies that confirmed glucose deprivation as a UPR inducer in several cell types [48,49].

UPR activation following ER stress has been found in pericytes [50], vascular endothelial cells, and retinal cells in several mouse models under chronic hyperglycaemic conditions [51]. Here, we showed augmented *Xbp-1* splicing, both in retinal explants after 48 h of low glucose conditions, and in ERAI mice subjected to a 5 h hypoglycaemic clamp in the retinas. As we showed that the GFP signal (which represents increased *Xbp-1* splicing in ERAI mice) partially co-localised with the staining for glial fibrillary acid protein (GFAP), we postulate that the Müller glial cells are activated under hypoglycaemic conditions. The role of Müller glial cells in diabetic retinopathy has been investigated for decades, and they are shown to be central to both neural and vascular viability [52].

GSH depletion has been shown to sensitize cells to apoptosis in a number of diseases, and sometimes thiol depletion alone is sufficient to trigger cell death [53,54]. To maintain an appropriate redox environment, the ER has its own pool of GSH, similar to other organelles such as mitochondria and nuclei. GSH biosynthesis is, however, restricted to the cytosol, and a transport system must be present in each of these organelles. Given the key role of GSH in ROS production, its quantification and uptake have been extensively investigated in mitochondria [55] and nuclei (for review, see [56]). In contrast, few studies exist on the quantification and transport of GSH in the ER [44,57], perhaps due to the difficulties in measurement of GSH/GSSG ratios in the ER. Therefore, the role of GSH_ER_ and GSSG_ER_ homeostasis remains controversial: ER flavoprotein Ero1, one of the most important drivers of disulphide-bond formation, seems to work properly independent of the GSH/GSSG ratio [43]. However, a recent study, performed in *S. cerevisiae*, has described Ero1 and Bip as regulators of the protein complex Sec61 which may control GSH transport to the ER, Ero1 activity being regulated by an indirect GSH reduction [57]. This result suggest that low glucose-induced ER stress may control GSH_ER_ concentration via Bip upregulation, this latter hypothesis need further investigation in mice.

In addition, several reports indicate a central role for GSH in ER homeostasis: protein disulfide isomerase (PDI), another key player in disulphide bond formation, seems to need GSH reducing power for proper functioning in vitro [58]. Moreover, depletion of GSH by BSO treatment has been shown to alter disulphide bond formation [59].

To test our hypothesis that GSH decrease is a player in ER redox disruption, we measured the GSH content in 661W photoreceptor-like cells under low glucose conditions, and determined UPR activation when GSH was decreased upon administration of BSO. We first showed a marked decrease in GSH after administration of BSO, indicating a link between cytosolic and ER GSH concentrations. However, despite the decrease in GSH levels observed in the ER after BSO treatment, the changes in UPR gene expression were not significant.

To further investigate the role of GSH in ER homeostasis in vivo, we used the *Gclm^−/−^* mouse model. These mice had 80% less retinal GSH content than their littermates (Figure 5). Consistent with previous studies that showed a correlation between GSH decrease and apoptosis in both hyperglycaemic [60] and hypoglycaemic conditions [33], these mice showed increased cell death in retinal explants in both hyperglycaemic (25 mM glucose concentration) and hypoglycaemic (1 mM glucose concentration) conditions after 24 h. When crossed with ERAI^tg/+^ mice, these mice maintained their ability to show increased *Xbp-1* splicing upon the induction of ER stress; however, they did not show any *Xbp-1* splicing in retinal sections. These results are in accordance with our in vitro results, and with the results of a study by Kritsiligkou et al., where a mutant mouse for thioredoxin reductase is susceptible to UPR independent of the GSH content [61]. However, due to the lack of characterisation of GSH exchange between the cytosol and ER in mammalian cells, further studies are needed to elucidate the role of GSH in the ER of retinal cells.

A great deal of literature suggests that UPR signalling could be targeted for therapeutic intervention in the degeneration of impaired retinal neurons. Reprogramming of UPR could slow the rate of retinal deterioration [62] and, in some cases, even result in long-term survival of retinal cells [63]. Moreover, there are studies that show UPR mitigation by the upregulation of the ER-resident molecular chaperone BiP in mice [17,18,64].

UPR is a mechanism of cell death involved in a number of diseases, including cancer and eye diseases, where neurodegeneration leads to blindness [65,66]. Neurodegeneration is an early event in the development of diabetic retinopathy and is an emerging research topic [67]. Promoting neural and photoreceptor survival may help delay the worst consequences of these diseases. Therefore, modulating UPR may promote neuroprotection and retinal cell survival. Although further studies are needed to investigate the role of UPR in retinal cell death under low glucose conditions, modulating UPR may offer a new therapeutic target to delay the worsening outcomes of diabetic retinopathy.

## Supplementary Materials

The following are available online at https://www.mdpi.com/article/10.3390/jcm10112529/s1. Figure S1: Tunicamycin (TU) injections increase *Xbp-1* splicing in ERAI mice. GFP expression was evaluated both in liver and retina by immunostaining after intraperitoneal (I.P.) injection (A). GFP expression was confirmed by Western blotting analysis performed on isolated retinas after intravitreal (I.V.) injection (B). Results are representative of 4 retinas for I.P injections and 8 retina for I.V injections. * *p* < 0.05. Figure S2: Muller glial cells are activated in ERAI^tg/+^ retinal explants after 48 h of exposure to low glucose concentration. GFAP staining on ERAI^tg/+^ retinal explants after 48 h of incubation with DMEM media containing 25 mM, 5 mM, and 1 mM glucose concentration. White, thin arrows show the colocalisation between GFAP staining and endogenous GFP signal, while head arrows show only GFAP staining and large arrows show only endogenous GFP. Results are representative of three retinal explants per condition. Table S1: PCR conditions.
